# Autologous stem cell-derived chondrocyte implantation with bio-targeted microspheres for the treatment of osteochondral defects

**DOI:** 10.1186/s13018-019-1434-0

**Published:** 2019-11-28

**Authors:** Murat Bozkurt, Mehmet Doğan Aşık, Safa Gürsoy, Mustafa Türk, Siyami Karahan, Berrak Gümüşkaya, Mustafa Akkaya, Mehmet Emin Şimşek, Nurdan Cay, Metin Doğan

**Affiliations:** 1 Department of Orthopedics and Traumatology, Ankara Yildirim Beyazit University, 06800 Ankara, Turkey; 2 Department of Medical Biology, Ankara Yildirim Beyazit University, 06800 Ankara, Turkey; 3 Deparment Of Bioengineering, Faculty of Engineering, Kırıkkale University, 72450 Kırıkkale, Turkey; 4 Deparment of Histology and Embryology, Faculty of Vetarinary Medicine, Kırıkkale University, 72450 Kırıkkale, Turkey; 5 Department of Pathology, Ankara Yildirim Beyazit University, 06800 Ankara, Turkey; 6 Department of Orthopedics and Traumatology, Ankara Yenimahalle Research and Training Hospital, 06800 Ankara, Turkey; 7 Deparment of Radiology, School of Medicine, Ankara Yildirim Beyazit University, 06800 Ankara, Turkey

**Keywords:** Cartilage, Microspheres, Bio-targeting, Tissue engineering

## Abstract

**Background:**

Chondral injury is a common problem around the world. Currently, there are several treatment strategies for these types of injuries. The possible complications and problems associated with conventional techniques lead us to investigate a minimally invasive and biotechnological alternative treatment. Combining tissue-engineering and microencapsulation technologies provide new direction for the development of biotechnological solutions. The aim of this study is to develop a minimal invasive tissue-engineering approach, using bio-targeted microspheres including autologous cells, for the treatment of the cartilage lesions.

**Method:**

In this study, a total of 28 sheeps of Akkaraman breed were randomly assigned to one of the following groups: control (group 1), microfracture (group 2), scaffold (group 3), and microsphere (group 4). Microspheres and scaffold group animals underwent adipose tissue collection prior to the treatment surgery. Mesenchymal cells collected from adipose tissue were differentiated into chondrocytes and encapsulated with scaffolds and microspheres. Osteochondral damage was conducted in the right knee joint of the sheep to create an animal model and all animals treated according to study groups.

**Results:**

Both macroscopic and radiologic examination showed that groups 3 and 4 have resulted better compared to the control and microfracture groups. Moreover, histologic assessments indicate hyaline-like cartilage formations in groups 3 and 4.

**Conclusion:**

In conclusion, we believe that the bio-targeted microspheres can be a more effective, easier, and safer approach for cartilage tissue engineering compared to previous alternatives.

## Introduction

Joint cartilage damage is a common problem around the world. Different epidemiological studies have suggested that the incidence rate of cartilage damage is about 60% [1–3]. The cartilage tissue is one of the main targets of tissue engineering due to its structural properties. Tissue scaffold applications are among the most successful surgical techniques for joint cartilage lesions. Recently developed biomaterials and scaffolds are giving hope to many patients with cartilage-related diseases.

When cartilage damage is detected, early treatment is vital for reducing symptoms, regaining functionality, and preventing the osteoarthritis development. There are a variety of surgical techniques available, from bone marrow stimulation to autologous chondrocyte implantation. Although, the discussion about which treatments are best for different patient groups is ongoing; currently, the most common surgical interventions are abrasion chondroplasty, bone marrow stimulation techniques (microfracture and drilling), mosaicplasty-osteochondral autograft transfer system (OATS), osteochondral allografts, and autologous chondrocyte implantation. Each treatment has advantages and disadvantages. Bone marrow stimulation techniques, such as microfracture, result in fibrous cartilage formation that is mainly composed of type I collagen. This is not the desired outcome compared to the hyaline cartilage formation and is not sufficient for long-term prevention of cartilage erosion and osteoarthritis [4, 5]. Mosaicplasty is the transfer of non-weight-bearing cartilage to the defect site. Although this one stage surgery results in better defect site recovery, the risk of donor site morbidity is a significant disadvantage [5].

Autologous chondrocyte implantation is one of the best alternative treatments as it results in hyaline-like cartilage formation. Although studies reveal histologically, morphologically, radiologically, and functionally improved results [6–10], the requirement of a two-stage and open surgery makes this technique less preferable. The possible complications of open surgery, including arthrofibrosis and infections, are driving surgeons to seek a new and/or a minimally invasive treatment alternative.

Emerging microencapsulation technologies can be used to leverage tissue-engineering technologies to create new treatment alternatives. Cell encapsulation technology is based on the immobilization of cells within a semipermeable membrane which protects the inner cells from both mechanical stress and the host’s immune system while allowing the bidirectional diffusion of nutrients, oxygen, and waste [11]. Specifically, there are many advantages of cell-encapsulated microspheres without which a targeted therapy option could not be created.

Articular cartilage is a unique tissue that is composed of a matrix and a limited number of cells. It has a limited repair capacity and is strongly affected by joint biomechanics [12–14].

The concomitant interaction of joint loading and extracellular matrix is necessary to maintain tissue integrity. Thus, cartilage tissue engineering needs animal modeling. The sheep knee joint is large enough to operate and resemble human knee joint in a greater degree [15].

The aim of this study is to develop a minimally invasive tissue-engineering alternative for the treatment of cartilage lesions by using microspheres that are targeted specifically to cartilage defects.

## Materials and method

This study includes both in vitro and in vivo experiments. The study protocol was approved by the Kırıkkale University Institutional Ethic Committee for Animal Experiments (protocol no: 13/13), and all methods were performed in accordance with the relevant guidelines and regulations. Adipose tissues were collected from animals and autologous mesenchymal stem cells (aMSC) isolated, cultured, and characterized in vitro. Then, isolated aMSCs were differentiated into chondrocytes and encapsulated into alginate microspheres. Alginate microspheres were coated with functionalized chitosan polymers, followed by antibody attachment to the surfaces of microspheres. These characterized bio-targeted microspheres were subsequently used in in vivo experiments.

### Stem cell isolation and characterization

One gram of adipose tissue samples was collected from animals under sterile conditions. Adipose tissues were washed three times with PBS. Then, tissues were cut into smaller pieces. Tissues were incubated at 37 °C for 120 min with 2 ml collagenase type I. Later, cells were transferred into a 15-ml falcon tube. After adding 5 ml PBS, the falcon tubes were centrifuged in 1800 RPM for 10 min. Following the supernatant removal, 5 ml of lysing solution was added to each tube and incubated at room temperature for 5 min. Later, 5 ml of PBS was added to incubated solution and the supernatant removed following centrifugation. These steps were repeated three times to give a better pellet. After, the final supernatant was removed, and pellets were pipetted into stem cell medium and cultured in 25 ml flasks. After reaching 70% confluence, cells were passaged and characterized with flow cytometer via CD44, CD45, CD90, and CD105 markers.

### Chondrocytes from aMSCs

For each animal subject, a flask of aMSCs were incubated in StemPro™ Chondrogenesis Differentiation Kit at 37 °C (Thermofisher Scientific, USA). Cell proliferation was examined daily, and after reaching 70–80% confluence, cells were passaged with tripsin-EDTA. Differentiated cells were characterized with immunohistochemical staining of type I collagen, type II collagen, and aggrecan on the 20th day.

### Immunocytochemistry

For this experiment, Horse Radish Peroxidase and DAB chromagen kits were used. On the 20th day of the culture, chondrocytes were transferred to slides and fixed with acetone. After fixation and PBS washing, %3 methanol hydrogen peroxide solution applied to the cells for 15 min. After PBS washing, protein blocking solution of HRP kit was applied to the cells. Cells were incubated with collagen type I, type II, and aggrecan antibodies for 1 h. Following to PBS washing, cells were incubated with secondary antibody and DAB chromogen kit.

### Microsphere and scaffold production

Microspheres were produced according to the methods of patent application PCT TR2017 050382. Cells were dispersed in 2% alginate to achieve 1 × 10^6^ cell per mm^3^ concentration. The capsuling device produced 500 μm microspheres via the addition alginate cell mixture drop wise into the calcium chloride solution. Likewise, 2% alginate solution was prepared and cross-linked with calcium chloride to produce scaffolds. Later, gels were lyophilized and seeded with 1 × 10^6^ cell per mm^3^ prior to the surgery. Both microspheres and scaffolds were made from alginate.

### Bio-targeting

Targeting was achieved by covering the alginate microspheres with functionalized chitosan polymers. Chitosan polymers were functionalized with biotin according to the biotinilation kit procedure. After washing three times, biotinilated polymers were sterilized under UV. The pH of the chitosan was then set to the 5.8 and added to the microspheres in CaCl_2_ solution. After a 15-min incubation, alginate microspheres were transferred into PBS for washing three times. Biotinilated chitosan covered alginate microspheres interact with avidinilated antibodies. The success of the bio-targeting process was evaluated by Fourier transform infrared spectrum FT-IR.

### Cell viability

Viability of the chondrocytes with and without capsules was tested by WST1. Ninety-six well plates including the same amount of cells were cultivated for 60 min in 37 °C in dark with the presence of WST1. Later, plates measured with a plate reader and cell viability percentages of the groups were calculated.

### In vivo experiments

A total of 28 female sheeps of Akkaraman breed were used in animal experiments. Their ages varied between 20 months and 22 months. Animals were kept in the sheep house of the Veterinary Faculty of Kırıkkale University with free access to water and fed twice a day and were free to walk around. All animals had a surgically created cartilage defect in the right knee joint. The animals were randomly assigned to one of the following 4 groups, each having 7 animals: control without any treatment (group 1), microfracture (group 2), cell-seeded scaffold (group 3), and microsphere (group 4). The food and water were withdrawn approximately 10 h before surgery. Animals went under general inhalation anesthesia with monitoring of vital health parameters. After skin shaving and povidone-iodine application, the right knee joint was opened with a longitudinal incision and then the joint surface was reached following a median parapatellar incision. A defect of 8 mm in diameter and 5 mm in depth was created in the weight-bearing area of the medial condyle of the femur. In control animals (group 1), the joint was closed without further procedure. During the surgery of the other three groups, the defect site was treated either with microfracture (group 2), cell-seeded scaffolds (group 3), or microsphere injection (group 4) according to groups they were assigned to [16–23].

### Post-operative care

During all parts of in vivo procedures and evaluation, the animals were under veterinary care and control. After the surgery of cartilage defect and subsequent post-operative care, all animals were examined twice a week. Various clinical and gait parameters, including general health status, joint inflammation, walking, weight loading to the joint, and running, were evaluated and recorded.

### Post-mortem evaluations

The post-operative follow-up was determined as 3 months like similar studies in the literature aiming cartilage regeneration with autologous cells [24–27].

Three months after the surgery of the cartilage defect and respective applications, animals were sacrificed under anesthesia. The right rear legs of the sacrificed animals were collected in toto and transferred to the Experimental Magnetic Resonance Imaging Center for post-mortem MR imaging (MRI). All of the MRI exams were performed with a 3 T MRI machine (Trio, Siemens, Erlangen, Germany) and 32-channel coil. During the MRI, the knee joint was in slight flexion and feet first-supine position. With MRI, two-dimensional (2D) T2-weighted (W) turbo spin-echo (TSE) (TR/TE 4000/71 msn, slice thickness(sth) 4 mm) images were obtained in the sagittal and coronal plane. Three-dimensional (3D) proton density W (3D-PDW) space (TR/TE 1200/32 msn, sth 0.5 mm) and 3D volume-interpolated breath-hold examination (3D-VIBE) (TR/TE 9.8/4.9 msn, sth 0.63 mm) images were obtained in the sagittal plane. T2 W trufi3D (TR/TE 8.8/3.8 msn, sth 0.4 mm), T2 3D short tau inversion recovery (T2-STIR) (TR/TE 5100/42 msn, sth 0.63 mm), and 3D-T2* mapping (TR/TE 422/11.32 msn, sth 0.63 mm) images were obtained in the coronal plane. For all 3D sequences, isotropic voxel sizes (< 1 mm^3^) were used and multiplanar reformatted images were also obtained. Field of view (FOV) was 15–16 cm, and matrix was 256–512/256–512.

In the MRI examinations, defect fill, cartilage interface, bone interface, surface, structure, signal intensity, subchondral lamina, subchondral bone, adhesion, and effusion were investigated. Modified magnetic resonance observation of cartilage repair tissue (MOCART) scoring was used to evaluate the cartilage tissue (maximum score 100) [28].

After MR imaging, the knees were carefully dissected to observe the healing. The joint was grossly examined, photographed, and evaluated according to the International Cartilage Repair Society cartilage repair assessment criteria [29]. In the evaluation process, the degree of defect repair, integration to border zone, and macroscopic appearance was used as parameters.

The articular defect site, including the subchondral bone beneath, was dissected avoiding any further damage and placed in 10% formalin for fixation. The 24-h formalin-fixed tissues were then placed in 4% formic acid solution for decalcification. Decalcified and trimmed tissue samples were dehydrated through alcohol series and embedded in paraffin blocks. The tissues in paraffin blocks were sectioned in 3-μm thickness for hematoxylin & eosin and toluidine blue staining. H&E and toluidine blue-stained tissue sections were subsequently evaluated for cartilage morphology, cellularity, and toluidine blue staining intensity according to the modified scoring system by Mankin et al. 1971 and analyzed with ANOVA.

## Results

### Bio-targeted microspheres carrying aMSC-derived chondrocyte

**Fig. 1:**
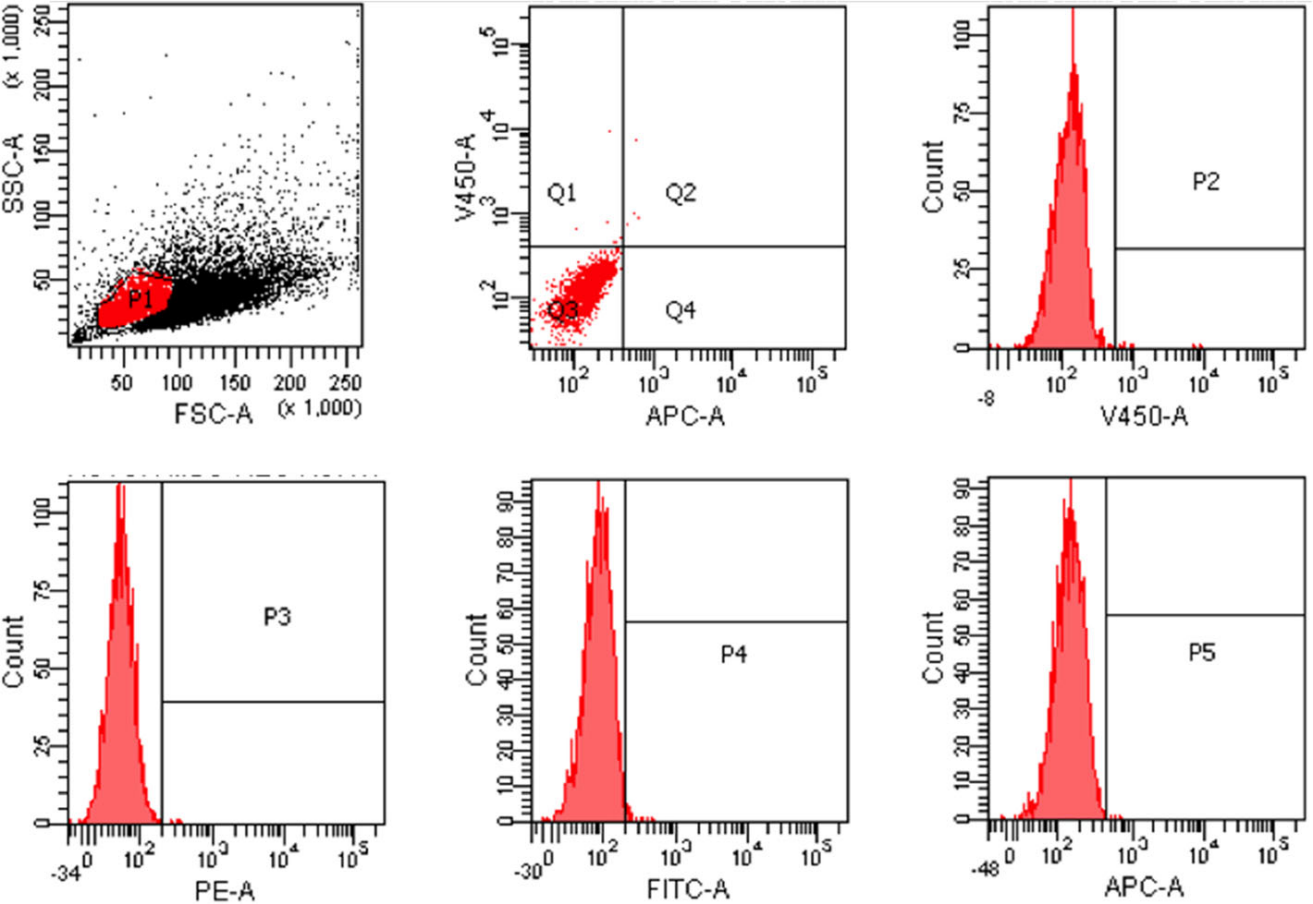
Flow cytometer results: negative controls

**Fig. 2:**
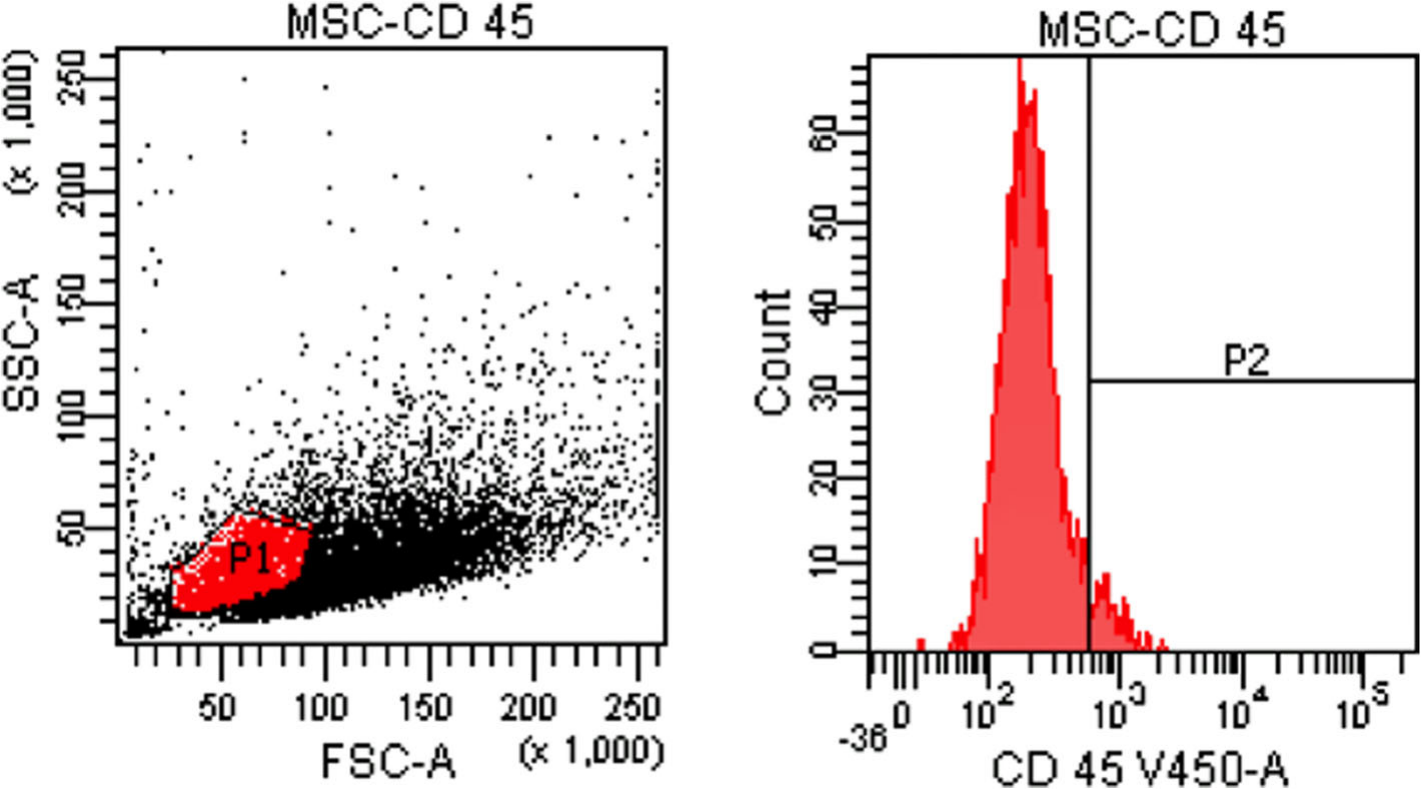
Flow cytometer results: CD-45-negative cells

**Fig. 3:**
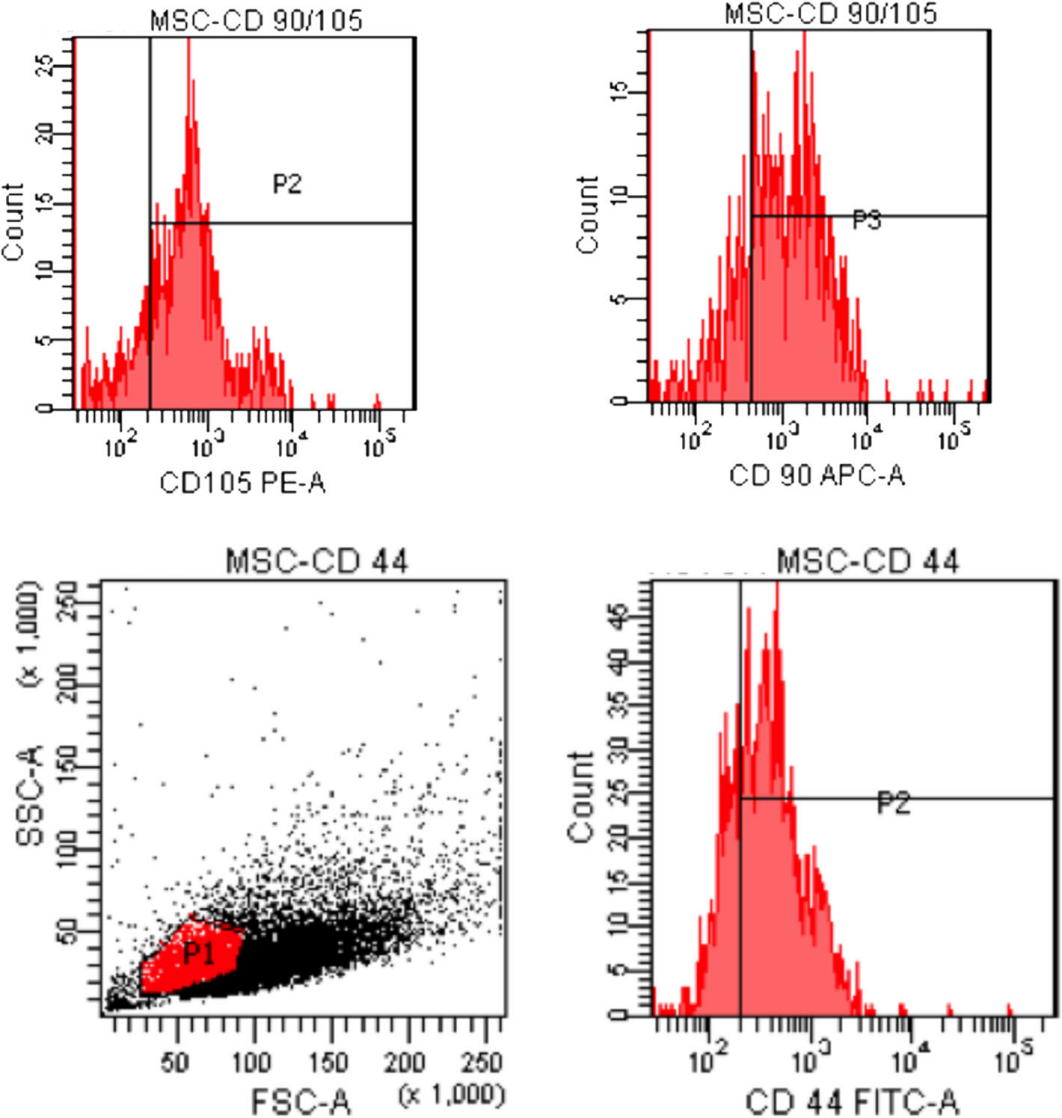
Flow cytometer results: CD-44-, CD 105-, CD 90-positive cells

Isolated stem cells were identified with flow cytometer. CD 45 negative (Figs. 1 and 2) and CD 44, CD90, and CD105 positive cells were accepted as the stem cells. Our results indicated that 70% of the cells were CD44, CD90, and CD105 (Fig. 3) positive.

**Fig. 4:**
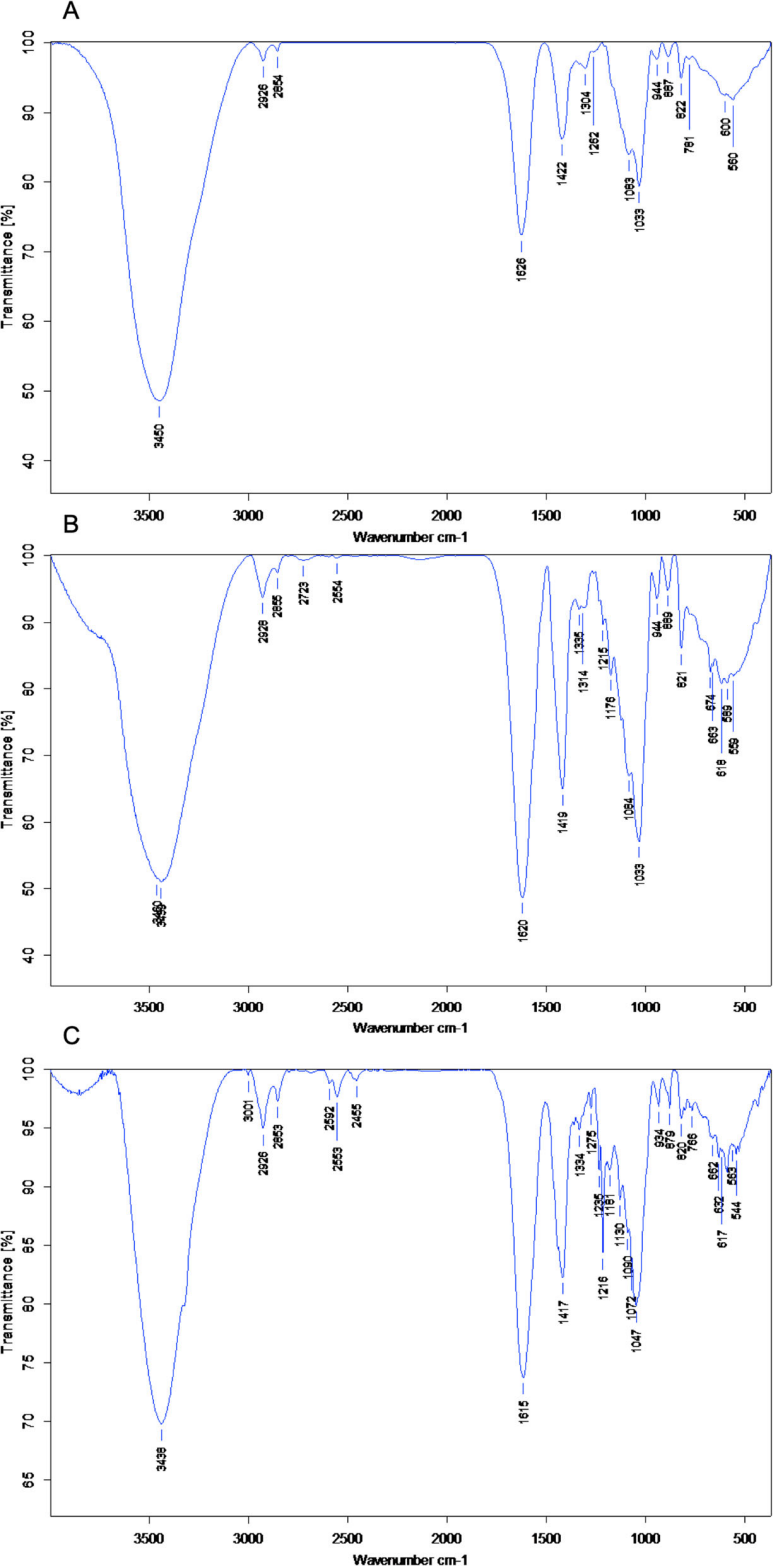
FT-IR results of **a** alginate microspheres. **b** Avidinilated linker-coated alginate microspheres. **c** Biotinilated antibody attached alginate microspheres

Before encapsulation, the targeting technic was evaluated by FT-IR. The results showed that alginate microcapsules could be covered by modified chitosan. Later, antibodies were attached to this modified surface in order to create a targeted microsphere (Fig. 4).

**Fig. 5:**
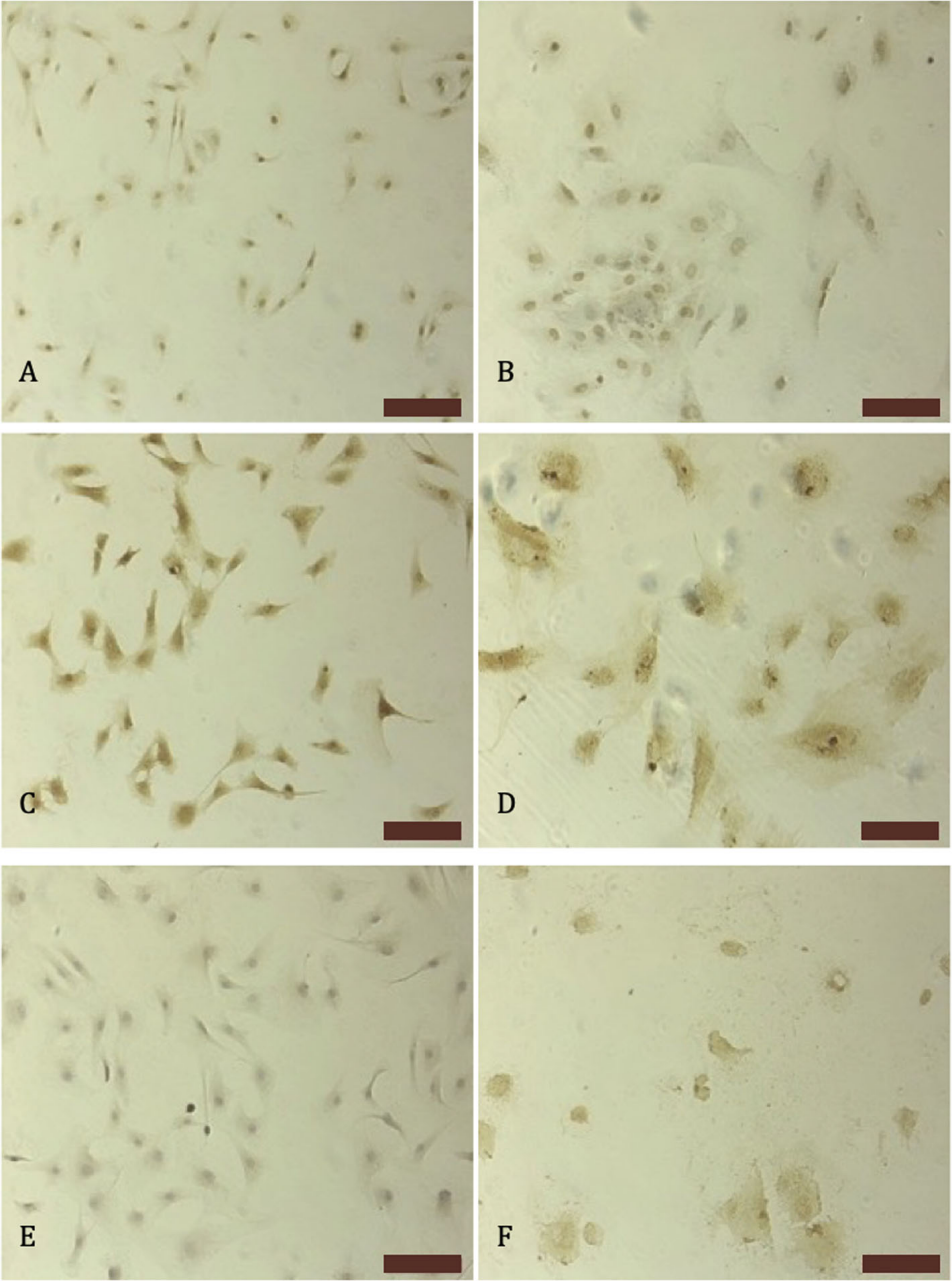
Immunohistochemical stainings on the 20th day of the culture collagen type I staining a stem cells b differentiated chondrocytes, collagen type II staining, c stem cells ddifferentiated chondrocytes, and aggrecan e stem cells f differentiated chondrocytes (bars100 μm)

**Fig. 6:**
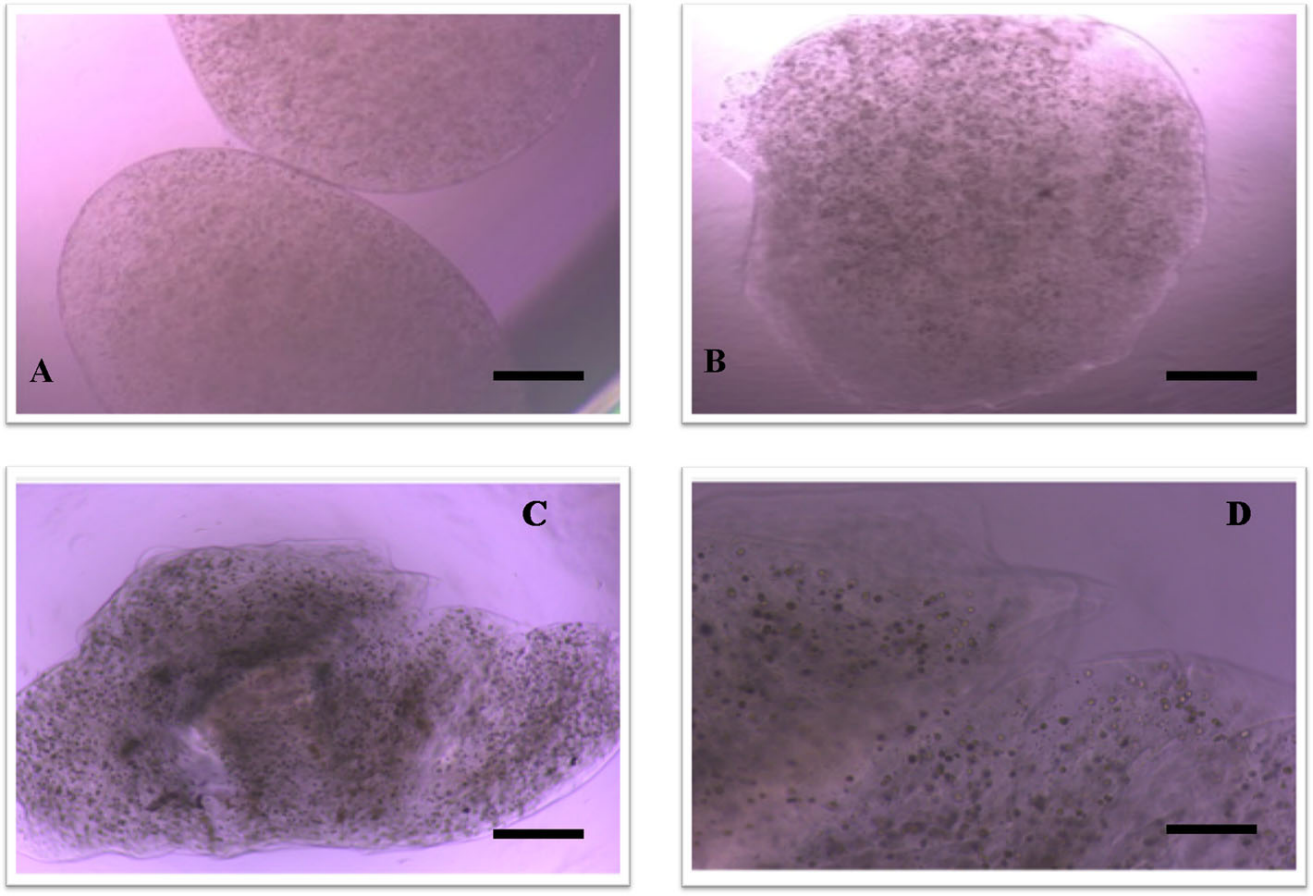
Microspheres under light microscope **a** 7th day × 20 (bar 100 μm), **b** 15th day × 20 (bar 100 μm), **c** 22th day × 20 (bar 100 μm), and **d** 22th day × 40 (bar 50 μm)

Stem cells were differentiated into chondrocytes with a high proportion (Fig. 5). The chondrocyte-encapsulated microspheres were intact during incubation for 3 weeks. After the 3rd week, microspheres started to degrade (Fig. 6). In Fig. 6, it can be seen that in vitro conditions microspheres started to degrade in PBS on the 15th day and on the 22nd day. This degradation increases in vitro. However, the animal experiment results indicate that the microspheres closer to the joint space degraded after 3 months. However, alginate microspheres cross-linked with Ca++ and due to the Ca++ release from the bone in vivo and the spheres closer to the subchondral bone had a slower degradation rates (Fig. 10d).

### Cell viability

Cell viability of the chondrocytes was investigated before and after encapsulation. The WST1 measurements of chondrocytes were accepted as 100. The encapsulated chondrocytes before targeting resulted in 62.23 ± 3.02, and the chondrocytes in the targeted microspheres resulted in 68.92 ± 3.37, which was similar to the scaffold viability reported in the literature [30].

### Clinical observations

Postoperatively, all the animals had a normal/healthy clinical appearance. No groups had problems with eating and drinking. Ten days after the surgery, the remaining sutures were removed. The redness at the operation site disappeared eventually. No indicators for local inflammation were observed at the surgery sites.

### MR imaging results

**Fig. 7:**
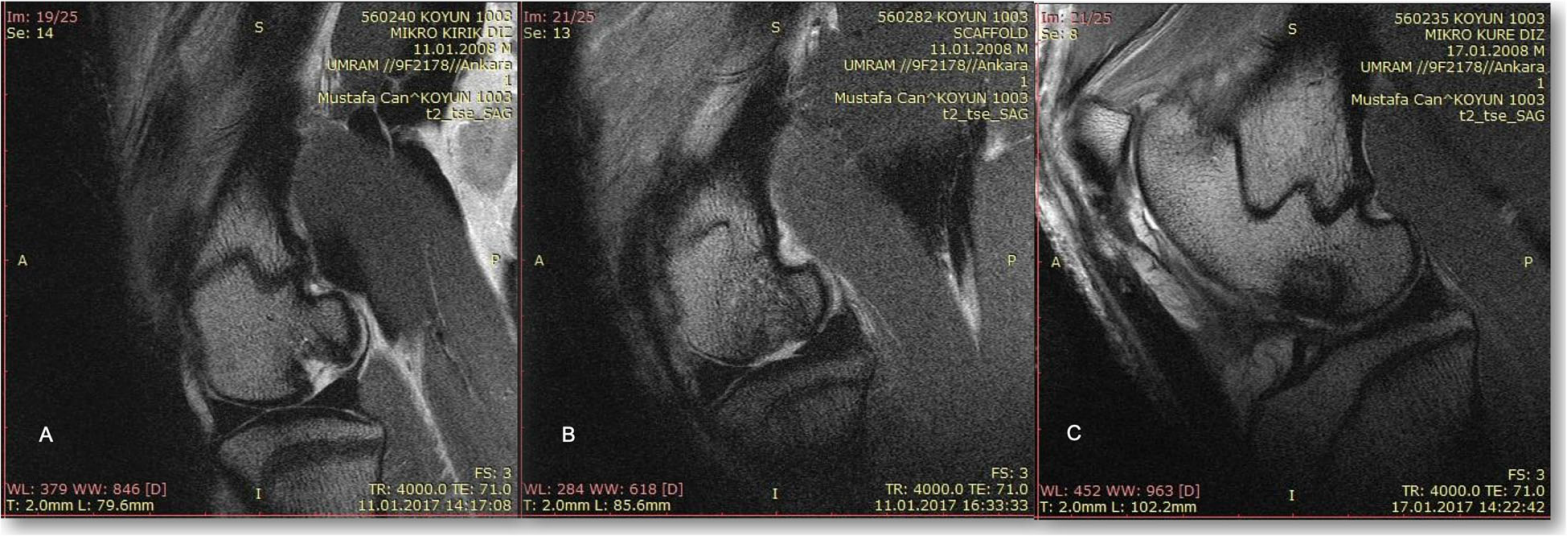
MRI evaluation of the cartilage repair: microfracture group (**a**), scaffold group (**b**), and microsphere group (**c**)

According to 3T MRI findings, mean modified MOCART scores were 11.86 in the control group, 32.71 in the microfracture group, 36.43 in the scaffold group, and 52.71 in the microsphere group. *P* values between control and the other three groups were < 0.001. There was no significant difference between microfracture and scaffold group (*p* = 0.323). The *p* values were 0.004 in between microfracture and microsphere and 0.015 in between scaffold and microsphere group, respectively (Fig. 7).

### Macroscopic examination

**Fig. 8:**
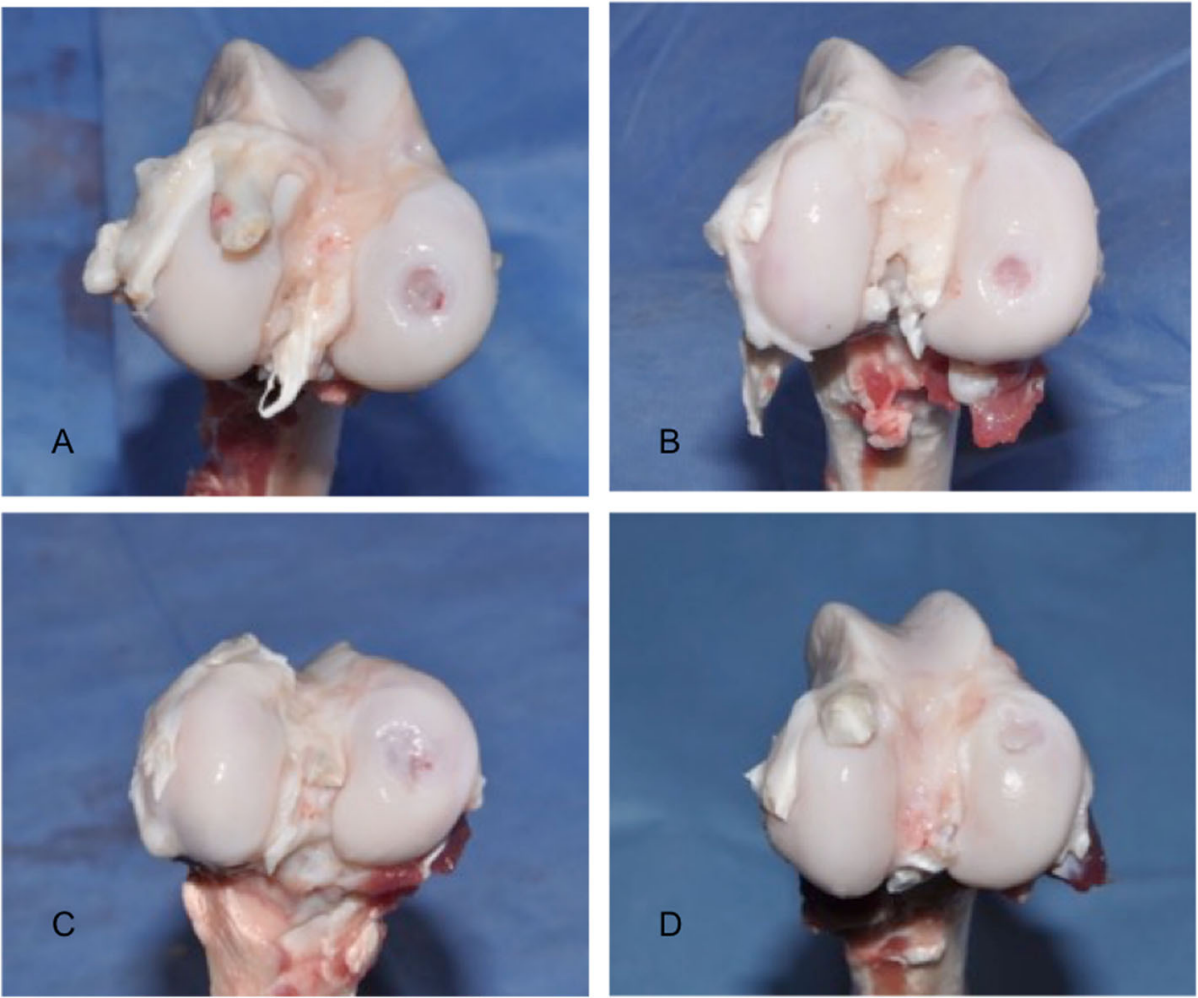
Macroscopic evaluation of the cartilage repair: control group (**a**), microfracture group (**b**), scaffold group (**c**), and microsphere group (**d**)

Using the International Cartilage Repair Society’s cartilage repair assessment criteria, scores for the regenerated tissues were as follows: control group (Fig. 8a) 2.7 (1–6), microfracture group (Fig. 8b) 6.6 (1–10), the scaffold (Fig. 8c) and microsphere (Fig. 8d) groups 8.3 (3–11) and 9.1 (5–11), respectively (Table 1).

**Table 1:** ICRS macroscopic evaluation of the repaired cartilages

Points	2.7 (1–6)	6.6 (1–10)	8.3 (3–11)	9.1 (5–11)
Grade	Grade IV	Grade III	Grade II	Grade II
Appearance	Severely abnormal	Abnormal	Nearly normal	Nearly normal

### Microscopic examinations

**Fig. 9:**
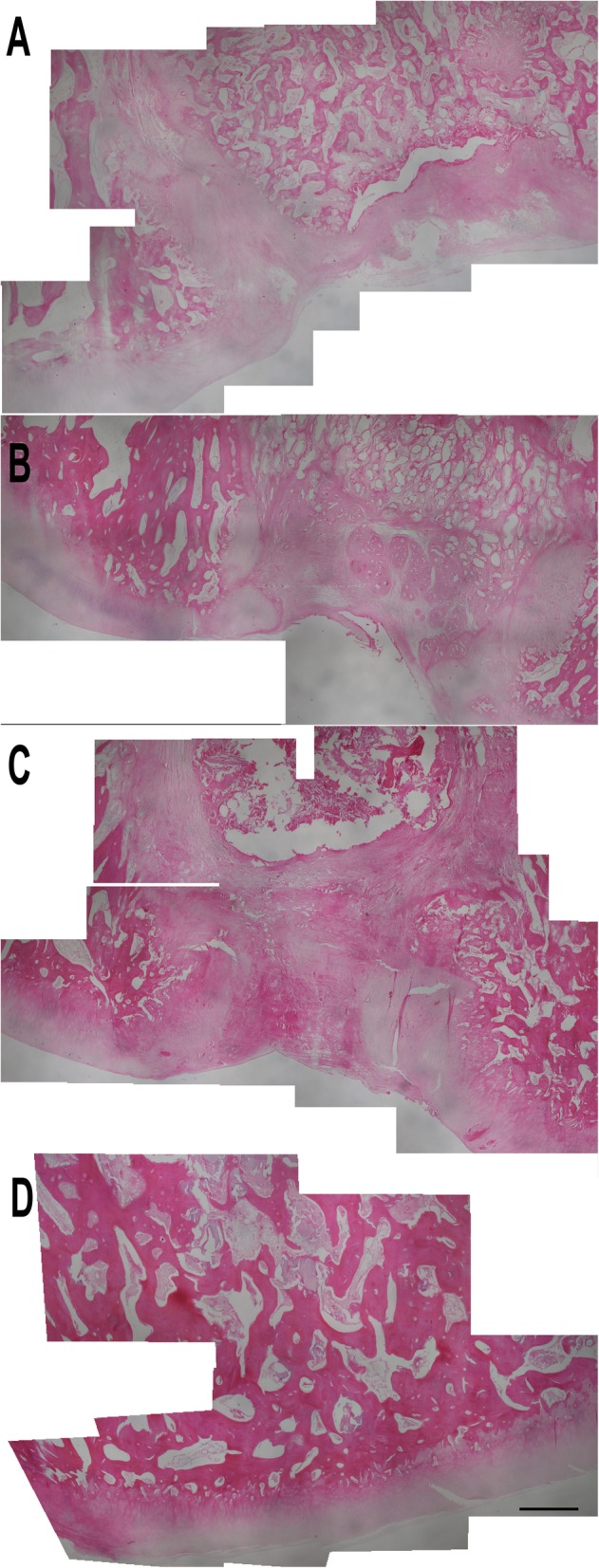
Microscopic assessment of the cartilage tissues at × 4 magnification with hematoxylin and eosin staining (bar 1000 μm): control group (**a**), microfracture group (**b**), scaffold group (**c**), and microsphere group (**d**)

H&E staining results (Fig. 9) showed that the control group had a scar tissue formation at the defect site. This tissue covered the defect, however, it included less cell compared to the normal cartilage tissue. The newly formed extracellular matrix could be observed as a thin cover over the defect.

The microfracture group examination reveals that fibrous cartilage tissue was formed in the defect site. However, the volume of the cartilage was not enough to fill up the defect. When compared to the other healthy sites of the articular cartilage, the amount of the recovery tissue was smaller in volume.

The scaffold group had a generally better outcome compared to the both control and microfracture groups. The recovery tissue filled almost the entire defect and integrated with the surrounding tissues. Like the scaffold group, microsphere group also filled the defect site with a regenerated tissue and this tissue was similar to the unaffected sites. Importantly, the cell count at the defect site was much higher when compared to all other groups.

**Fig. 10:**
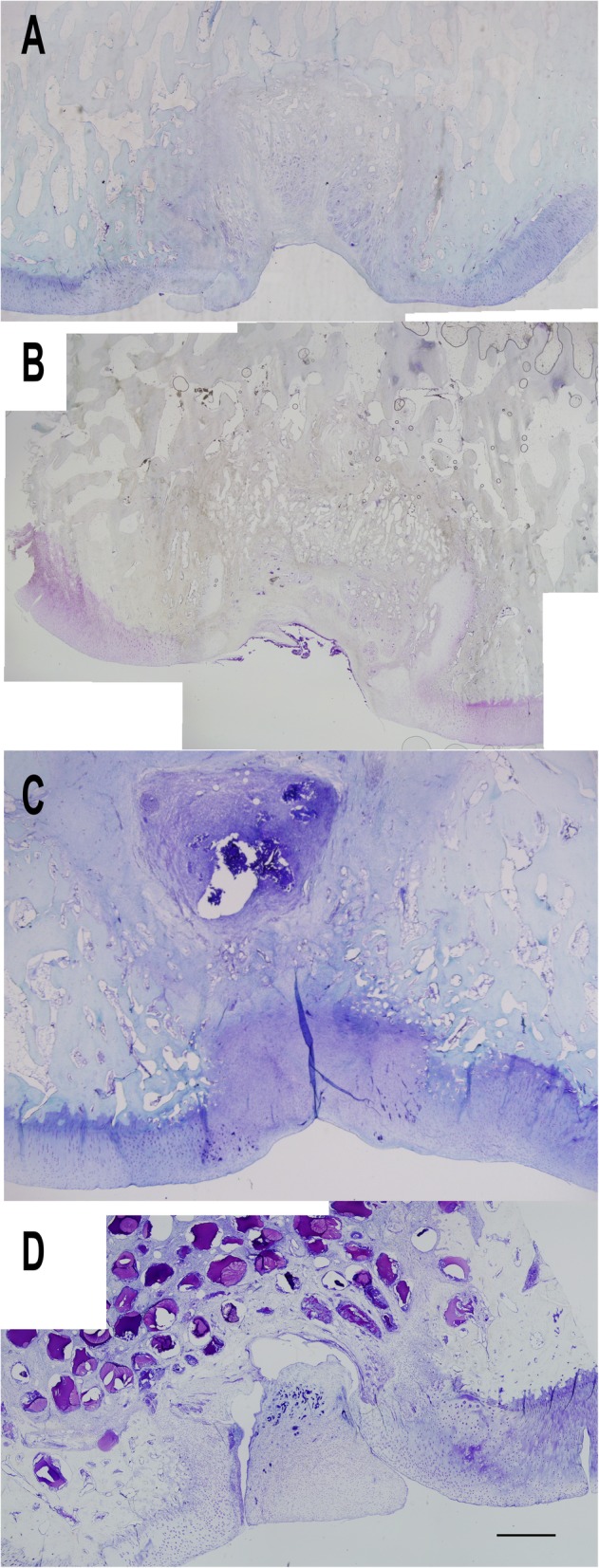
Microscopic assessment of the cartilage tissues at × 2 magnification with toluidine blue staining (bar 100 μm): control group (**a**), microfracture group (**b**), scaffold group (**c**), and microsphere group (**d**)

Toluidine blue staining (Fig. 10) results showed a similar pattern to the H&E staining and macroscopic findings. In the control group, the scar tissue had a reduced toluidine blue staining. This indicates that the recovery tissue was fibrous tissue and not a cartilage tissue.

Although, in the microfracture group, the staining intensity indicated that some cartilage tissue was formed and the recovery tissue was mainly made up of fibroblasts and fibrous cartilage. Compared to healthy cartilage, the recovery tissue was lacking the subchondral junction/tidemark and located just over the bone tissue.

As opposed to these histomorphologic findings, the recovery tissue was partially hyaline-like and partially fibrous cartilage in the scaffold group. Although, the cells were scattered throughout the recovery tissue, and the hyaline-like cartilage formation was sparse. On the other hand, there were more hyaline-like formations in the microsphere group. Also, there was formation of mineralized tissue between the bone and cartilage. The cell counts, cell alignments, and cell distributions were more similar to the healthy tissue compared the other groups.

According to modified Mankin cartilage assessment criteria, scores of the regenerated tissues were as follows: control group (Fig. 10a) 9.8, microfracture group (Fig. 10b) 8.33 (1–10), scaffold (Fig. 10c), and microsphere (Fig. 10d) groups 4.86 and 4.33 (*p* = 0.024 between the groups), respectively.

## Discussion

In this study, a minimally invasive tissue-engineering approach, in which specific proteins of the cartilage were used as targets for antibody-coated microspheres, was created for cartilage defect treatments. Ideal treatments should result in hyaline-like cartilage formation filling up the defect site and an integrated recovery tissue with good mechanical properties.

There are many studies showing and comparing the results of autologous chondrocyte implantation (ACI). These studies indicate that ACI treatment has a positive effect on pain, activity, and functionality according to Lysholm-Gillquist scale [6–8, 31–35], Tegner-Lysholm scale [8, 31–34], VAS pain score [6, 7, 32], International Knee Documentation Classification (IKDC) [8, 32–34] scale, and American Orthopaedic Foot and Ankle Society clinical function scale [36]. Arthroscopic patient examinations demonstrated that healing tissue was strong, stable, and integrated with surrounding tissues. Moreover, these studies consist of MR images which validate restored cartilage, filled defect, and integration with the surrounding tissue [6–8]. Furthermore, histology examination 6 months after the ACI application proves that newly formed cartilage had hyaline-like properties [9, 10] and following repeated biopsies showed that the amount of the hyaline cartilage increased [9].

However, ACI is a two-stage open surgical procedure and this presents difficulties from clinical perspective. In addition, these two stages including the cartilage sample being taking out and the implementation of the cell-seeded scaffold which are both invasive techniques and require open surgery. The main reason that this surgery remains rare is these difficulties. The preference of surgeons is for less invasive techniques, such as an injectable or arthroscopy-assisted one-stage treatment. Although, there are some one-stage techniques that result in fibrous cartilage formation, and these are less preferable due to the weak mechanical properties of collagen type I [37, 38].

Our study indicated that the designed injectable treatment resulted in a similar yet slightly better tissue regeneration compared to cell-seeded scaffold application that requires open surgery. When microscopically examined, H&E and toluidine blue staining indicate similar results with macroscopic analyses (Fig. 6). In the control group, there is a thin layer of scar tissue formation, but it lacked cartilage cells and matrix. On the other hand, the microfracture group also resulted in fibrous tissue formation with more chondrocytes but without cartilage matrix properties. The other two groups including cellular treatment filled the defect site better as expected. The cause of these better results could be the presence of polymer containing extracellular matrix-like structure in the treatments.

When cellular treatment group results were compared, there was a small difference in macroscopic analyses (8.3/9.1, group 3/4 respectively), but the histomorphological difference was conspicuous. Especially, the cell distribution and/or alignment in scaffold was irregular and showed both fibrocartilage and hyaline-like cartilage formation in different areas. As mentioned before, there are studies showing hyaline cartilage 6 months after treatment. Our results may lack the required time period, because of the 3-month study design. However, the microsphere group had a better cell distribution and/or alignment. The healed cartilage was more hyaline-like (Fig. 9) in microsphere group. Moreover, in the integration site of microspheres, it was observed that cells had a tendency to align parallel to the surface closer to the synovia, mimicking the normal counterpart.

Microspheres have started to earn their place in biomedical applications including tissue engineering [39]. The bone tissue-engineering study of Chan et al. is an example of these tissue-engineering applications [40]. Later, microspheres have been applied to the nerve, liver, heart, and skin tissues and it has been shown that healing is related to the microsphere production method, microsphere size, and application method [41]. On the other hand, there are many studies showing that nanoparticles can be bio-targeted to a damaged or diseased tissue in vivo*.* These studies mostly focused on the delivery of therapeutic agents such as drugs or genetic materials. Although in these bio-targeting studies, intravenous microsphere application is avoided due to the capillary blockage risk, and there are many studies that use microspheres as drug release tools. In our bio-targeting study, since the treatment is applied intraarticularly, the application site does not have a risk of capillary blockage. Microspheres attach specifically to the defect site and to each other in order to create an extracellular matrix-like structure in situ.

In an animal model study, application with cells—compared to without cells—resulted in 18% better healing. Also, it is stated that addition of the cell, as an important component of tissue engineering, leads to healing by production of many bioactive components [42]. A meta-analysis including 117 studies by Pot et al. indicated that although results varied between different cell sources, the results of the applications including cells were superior to application without cells [43]. As also stated by an important author of autologous chondrocyte implantation Brittberg, cells are always necessary to repair a damaged tissue in terms of biology [44]. The groups of this study designed under the light of this information only include treatments with cells which were shown to be superior compared to the alternatives without cells.

This study lacks the proofs of the microsphere movement inside the joint and how they reach the targeted area. The other question is whether these microspheres interact with other tissues. Moreover, in this study, only one formulation for microspheres/polymer ratio was tested, and other ratios may give a better cartilage recovery. For this reason, further studies should include a transparent bioreactor for ex vivo experiments of cartilage defects. In this design, it would be possible to observe the behaviors of the microspheres, attachment of the microspheres to the defect site, and assembly of spheres to form a scaffold. Also, this would be an effective way of testing different formulations of treatment.

In conclusion, in vivo results indicate that bio-targeted microsphere application is a good candidate for cartilage defect treatment. Specifically, the minimally invasive application procedure that was investigated in our paper appears superior to open surgical treatments. Although, cell-seeded scaffold results and microsphere results were similar, and the methods of applications are incomparable: injection vs open surgery.

## Data Availability

Not applicable.

## Abbreviations

2D: Two-dimensional
3D: Three-dimensional
ACI: Autologous chondrocyte implantation
aMSC: Autologous mesenchymal stem cells
FT-IR: Fourier transform infrared spectrum
H&E: Hematoxyline & eosin
IKDC: International Knee Documentation Classification
MOCART: Modified magnetic resonance observation of cartilage repair tissue
MRI: Magnetic resonance imaging
OATS: Osteochondral autograft transfer system
